# A Case-Control Study of the Role of Human Papillomavirus in Oesophageal Squamous Cell Carcinoma in Australia

**DOI:** 10.1155/2014/236482

**Published:** 2014-04-28

**Authors:** Surabhi S. Liyanage, Eva Segelov, Aisha Malik, Suzanne M. Garland, Sepehr N. Tabrizi, Eleanor Cummins, Holly Seale, Bayzidur Rahman, Aye Moa, Andrew P. Barbour, Philip J. Crowe, C. Raina MacIntyre

**Affiliations:** ^1^School of Public Health and Community Medicine, The University of New South Wales, Kensington, NSW 2052, Australia; ^2^Department of Medicine, St. Vincent's Hospital Clinical School, The University of New South Wales, Kensington, NSW 2052, Australia; ^3^UNSW Medicine, The University of New South Wales, Kensington, NSW 2052, Australia; ^4^Department of Microbiology and Infectious Diseases, The Royal Women's Hospital, Parkville, VIC 3052, Australia; ^5^Department of Obstetrics and Gynecology, Murdoch Children's Research Institute, University of Melbourne, Parkville, VIC 3010, Australia; ^6^Department of Surgery, Princess Alexandra Hospital, The University of Queensland, St Lucia, QLD 4072, Australia; ^7^Department of Surgery, Prince of Wales Hospital Clinical School, The University of New South Wales, Kensington, NSW 2052, Australia

## Abstract

*Objective*. We investigate the prevalence of human papillomavirus (HPV) in oesophageal squamous cell carcinoma (OSCC) tissues compared to oesophageal tissue from healthy controls, in an Australian cohort. *Methods*. We conducted a hospital-based case-control study of 99 patients with OSCC and 100 healthy controls to examine the presence of HPV DNA. Paraffin tissues were tested using the PapType high-risk HPV detection and genotyping kit and with INNO-LiPA HPV Genotyping Extra. The biopsy samples were tested for HPV using a PCR-ELISA method based on the L1 consensus primer set PGMY09-PGMY11. *Results*. HPV DNA of the oncogenic genotype 16 was detected in 1/99 case specimens, a rate of 1010 per 100,000 (95% CI: 30–5500). All control specimens were negative for HPV. Significantly higher rates of smoking, other aerodigestive cancers, and mortality were seen among cases than controls. A pooled analysis of this study and the only other Australian case-control study found that 9/321 cases and 0/155 controls were positive for HPV. The pooled odds ratio for HPV being a risk factor for OSCC was 9.35 (95% CI: 0.47–190.33). *Conclusion*. Our results suggest that in this multifactorial cancer HPV may be an additional risk factor; although a larger, better powered study is needed.

## 1. Introduction

Oesophageal cancer (OC) is currently the sixth most common cause of cancer-related death in the world, claiming the lives of 406,000 people in 2008 [1]. It is two to four times more common in males than females and is the eighth most common malignancy globally, with an annual worldwide incidence of nearly half a million cases [1]. The majority of OCs are divided into two histopathological categories: adenocarcinoma, predominant in the Caucasian population and usually occurring in the lower third of the oesophagus, and squamous cell carcinoma, more frequent in Asians and Africans and generally found in the upper and middle thirds of the oesophagus [2, 3]. In the last three decades, the incidence rate of oesophageal squamous cell carcinoma (OSCC) in Western countries has remained stable and has been overtaken by the relative rise in incidence of adenocarcinoma [4, 5]. However, OSCC is still the predominant histologic subtype of OC worldwide [5].

The multifactorial aetiology of OSCC may explain its highly variable geographical distribution with up to a 500-fold difference in incidence between high-risk areas such as the Transkei region of South Africa, the Caspian Littoral of Iran, and Northern China and low-risk regions such as Western Africa [1, 6]. On average, the incidence rate of OSCC in most countries is from 2.5 to 5 per 100,000 cases for males and from 1.5 to 2.5 per 100,000 for females [7]. Australia is categorised as a low-risk country for OSCC [8]. In low-risk OSCC regions such as Australia and the United States, 9 out of 10 OSCC cases are thought to be related to excess alcohol consumption and tobacco smoking [9]. In the regions of the highest risk, approximately 90% of oesophageal cancers are SCCs [10]. Although the pathogenesis of OSCC in high-risk regions remains unclear, some of the aetiological factors thought to be responsible are thermal damage to oesophageal mucosa from ingestion of food and beverages at high temperatures [11, 12], poor nutrition including low intake of fruits and vegetables, increased consumption of processed and red meat, pickled and preserved foods [13, 14], exposure to nitrosamines [15] and certain infectious agents, in particular, human papillomaviruses (HPV) [7].

The International Agency on Research on Cancer (IARC) has acknowledged HPV involvement in head and neck, particularly oropharyngeal, tonsillar, and oral SCC, but has not yet made a conclusive statement about a causal relationship between HPV and OSCC [16]. Syrjanen et al. first suggested a possible link between HPV and oesophageal squamous cell papilloma (OSCP) following observations of characteristic cytopathic changes of HPV infection usually seen in condylomatous lesions, in both benign oesophageal epithelial tissue and malignant oesophageal tumours [17]. Syrjanen's postulate was validated by studies which used immunohistochemistry to detect HPV structural proteins in oesophageal lesions [18, 19]. Case-control methodology testing for the presence of HPV DNA in OSCC tissue compared to oesophageal tissue from healthy subjects is the most suitable study design to investigate any potential HPV-OSCC link. To date, there have been approximately twenty-six small-scale case-control studies carried out in this area, along with more than hundred case-series reports of varying conclusions [20]. We have recently shown in a meta-analysis of published case control studies that there is a strong association between HPV and OSCC [21]. We also showed for the first time that this association is greater for low- and medium-risk countries than for high-risk countries [21].

The inconsistent observations between individual studies may be attributed to many factors including differences in study design, laboratory techniques, sample types, testing methodology, and the dynamic aetiology of OSCC [20]. A total of three studies including one case-control and two case series have been carried out in Australia from 1986 to date to investigate a potential association between HPV OSCC tissues [22–24]. They have produced mixed results, although the authors of the previous case-control study concluded that HPV is not a risk factor for OSCC [22]. We aimed to determine whether HPV is associated with OSCC in Australia.

## 2. Methods

### 2.1. Study Design and Setting

We conducted a case-control study to investigate whether HPV is associated with OSCC in Australia. Case specimens were obtained retrospectively from tumor banks at five hospitals nationwide. These included three teaching hospitals in New South Wales, one in Queensland, and a cancer hospital in Victoria. Control subjects were recruited prospectively in 2010, at the endoscopy clinics of two of the hospitals in NSW.

### 2.2. Selection of Cases and Controls

Case subjects were defined as patients who had a histopathologically confirmed diagnosis of OSCC. Five tumour banks across the country were approached and any OSCC specimens available were requested and obtained. Case specimens included 23 genomes amplified, 26 fresh frozen, and 50 formalin-fixed and paraffin-embedded samples. Formalin-fixed and paraffin-embedded samples were reviewed by a pathologist to ensure that appropriate sections were obtained for testing. Case specimens used in this study dated from 1989 to 2009.

Controls were defined as healthy subjects with no previous history of gastric or oesophageal malignancy and no underlying chronic illness. In addition, those with any history of antecedent or concurrent precursor lesions for adenocarcinoma, such as Barrett's oesophagus, were excluded from recruitment. Patients who were not suitable for oesophageal biopsy, such as those with a predisposition to bleeding, were also excluded. Subjects were randomly sampled from patients undergoing routine endoscopy for investigation of presumed nonmalignant conditions such as gastrooesophageal reflux disease. Areas of oesophagus biopsied were macroscopically normal in appearance. Three biopsies were obtained from the upper, middle and lower thirds of the oesophagus, for each control.

Of a total of 126 controls who were approached, two were excluded due to a previous history of Barrett's oesophagus, three were excluded due to concurrent warfarin therapy which contraindicated biopsy during procedure, and twenty-two people declined invitation to take part in the study.

### 2.3. HPV Detection Lab Testing

A range of commercial and in-house adapted HPV detection methods were utilized to test all case and control specimens. All testing was carried out at the HPV Labnet, the World Health Organization Western Pacific regional HPV reference laboratory at the Royal Women's Hospital, Victoria, Australia.

For paraffin-embedded archival samples, two commercially available assays utilizing PCR and targeting 65 and 150 bp region of L1 were used. The smaller amplicons are particularly suited for archival tissue [25–27]. 5–10 mm sections of FFPE archival tissue were deparaffinised with 800 µL histolene then mixed with 400 µL absolute ethanol. The tissue was centrifuged at 14,000 ×g for 2 minutes. The resultant pellet was washed with 1 mL of absolute ethanol and centrifuged for 2 minutes and the supernatant was discarded. The pellet was air-dried and subsequently incubated for 4 hours at 55°C with 160 µL tissue lysis buffer (Roche Diagnostics, Mannheim, Germany) and proteinase K at a final concentration of 1 mg/mL. DNA was then isolated from digested tissue using the MagNA Pure LC DNA isolation kit I (Roche Diagnostics) on the automated MagNA Pure LC extraction system, according to the manufacture's protocol, with the addition of 33.3 mg/mL of poly(A) RNA carrier to the lysis buffer which has been demonstrated to improve extraction efficiency. Nucleic acid was eluted into a final volume of 100 µL.

Fresh biopsy samples were tested using well-established PGMY09/11 primers either in a PCR-ELISA format as described previously [28, 29] or on Roche HPV Linear array. Fresh 1-2 mm biopsies were homogenised using MagNA Lyser (Roche Diagnostic) with 250 mL of tissue lysis buffer (Roche Diagnostics), by two repetitions of 30 seconds at a speed of 7000 rpm in the MagNA Lyser Instrument. Proteinase K was added to a final concentration of 1 mg/mL, and samples were incubated at 55°C until fully digested. DNA was then isolated from the digested tissue as described above. DNA was isolated from the PreservCyt samples of cervical cells using the MagNA Pure LC DNA isolation kit I on the automated MagNA Pure LC extraction system.

Nucleic acid extracted from FFPE was tested using the PapType high-risk HPV detection and genotyping kit (Genera Biosystems, Melbourne, Australia) and with the INNO-LiPA HPV genotyping extra (INNO-LiPA) (Innogenetics, Ghent, Belgium) as both methods amplify a relatively small gene region and are preferred for samples that may be degraded or contain low levels of DNA [27]. Both methods were performed according to the manufacturer's instructions.

PapType detects 14 high-risk and 2 low-risk HPV genotypes using fluorescently labelled primers to simultaneously amplify a 150 base-pair (bp) region of the HPV L1 gene and includes a 288 bp region of the human-specific gene, myosin light chain (MLC-1) as an internal control. PCR product is hybridized to HPV type-specific probes bound to labelled silica microspheres. These detection beads are distinguishable by their size and fluorescent intensity using flow cytometry (BD FACSArray Bioanalyzer, Becton Dickinson, Franklin Lakes, New Jersey, USA) and the data interpreted using QPlots (Genera Biosystems).

InnoLipa is a multiplex PCR-based assay (SPF_10_-modified primers), followed by reverse line blot hybridization, detects and genotypes 28 different HPV genotypes, including 18 high-risk HPVs (16, 18, 26, 31, 33, 35, 39, 45, 51, 52, 53, 56, 58, 59, 66, 68, 73, and 82), 6 low-risk HPVs (6, 11, 40, 43, 44, 54, and 70), and 3 other non-classified HPVs (69, 71, and 74), targeting a 65 bp fragment of the HPV L1 ORF. DNA and WGA DNA samples were tested using PapType as described above, because DNA volumes were limited.

Nucleic acid from biopsy samples and whole genome amplified DNA were amplified using HPV L1 consensus primer set PGMY09-PGMY11 [28]. PCR products were detected by ELISA using biotin-labeled probes designed to detect mucosal HPV sequences in the sample [29].

Any biopsy samples that tested positive for HPV by either PapType or the PGMY09-PGMY11 PCR-ELISA were subsequently tested using the LINEAR ARRAY HPV Genotyping Test (Roche) with the modification of using an automated blot processor, BeeBlot (Bee Robotics Ltd., Gwynedd, United Kingdom), for the hybridization and washing steps, as previously validated by the Royale Women's Hospital laboratory [30–32].

In addition to being tested by Linear Array using 50 µL of DNA as per manufacturer's instructions, these samples were also tested using 10 µL DNA (plus 40 µL water) to minimize the potential effect on PCR competition by an overabundance of human genomic DNA in the sample. Both high *β*-globin and low *β*-globin controls must have been visible for the strip results to be considered valid for that sample.

### 2.4. Sample Size and Data Collection

Based on available literature, we estimated the presence of HPV DNA to be at least 15% in cases and less than 3% in controls. To detect this difference with 95% confidence and 80% power, 55 subjects per arm are required. We therefore aimed to recruit 100 cases and 100 controls. Due to the small number of annual OSCC cases across Australia, all cancer samples included in this study were archival specimens from tumor banks.

Data collection for cases included retrieval of information primarily from hospital medical records and, where possible, direct patient contact via telephone interviews. One investigator (SL) reviewed medical records and collated the various sources of data for OSCC cases. Detailed data collection for case subjects was not possible for several reasons. Many of the OSCC patients, whose tissues were used in our study as cases, were deceased at the time this study was conducted and therefore information could only be obtained from hospital medical records and patient files from the consulting rooms. In addition, some hospitals had destroyed medical records of patients whose admissions had occurred more than 10 years ago. Several patients had been treated at multiple medical facilities across the state and the country and internationally, at various stages of their illness, thereby making it difficult to obtain a complete record. Finally, some OSCC patients either were not contactable or declined invitations to participate in a phone interview. In contrast, all relevant information was collected prospectively for each of the 100 control subjects during their visit to endoscopy clinics. Control data were collected prospectively prior to oesophageal biopsy by an investigator-administered survey by two investigators, SL and AM.

### 2.5. Data Analysis

As the data collection for cases was retrospective (and many cases were deceased) and that for controls was prospective, there was a substantial difference in the quality and quantity of information obtained between cases and controls. As a result of the inconsistency and incompleteness of data in cases compared to control subjects in the study, data analysis for other risk factors (such as smoking, alcohol, and diet) for OSCC was not possible. Therefore, this is mainly a descriptive study concentrating primarily on prevalence of HPV DNA in OSCC compared to normal oesophageal tissue. *P* values were calculated using the Epi Info 3.5.5 software program [33].

OSCC is a rare cancer in Australia, and collection of control specimens entails an invasive oesophageal procedure on healthy subjects. As such, large studies are unfeasible. In order to improve the statistical power, a pooled analysis was conducted combining the results of the only other Australian case-control studies [22], which found 8 out of 222 HPV positive cases and no HPV in 55 normal oesophageal tissue controls. The data required for this analysis were available in the published paper. Combined data from the two case-control studies resulted in 321 cases and 155 controls. We used the penalized likelihood method to estimate the pooled odds ratio (OR) from logistic regression. The confidence integral of the OR was estimated using profile likelihood estimate [34]. This analysis was done with SAS 9.3, using PROC LOGISTIC with “FIRTH” option in the model statement and using “CLPARM=PL” statement.

### 2.6. Ethics Approvals

Ethics approval for this study was obtained from the lead ethics committee at Northern Sydney Central Coast Health in Sydney, Australia. In addition, ethics approval was obtained from each of the five sites of specimen collection. All participants in the study were provided with printed information regarding the study and written consent was obtained prior to recruitment. Reading material provided to recruits pertaining to the study and all patient data collection forms were approved by the Lead Ethics Committee.

## 3. Results

Table 1 describes the demographic and basic characteristics of cases and controls. The control subjects ranged from 16 to 84 years of age, with a mean age of 52.3 years. The youngest OSCC patient was aged 39 at time of diagnosis, while the eldest was 92 years old. The mean age of case subjects at time of diagnosis was 67.5 years. Gender distribution between cases and controls was equal in the study.

Two-thirds (65%) of case subjects smoked during their lifetime compared to 45% of controls (*P* < 0.01). Case (OSCC) subjects also had a higher rate (17%) of past aerodigestive cancers, including hypopharyngeal and tonsillar SCCs, while none of the control subjects had a past history of malignancy (*P* < 0.001) as per our selection criteria. Thirty-nine of the ninety-nine case subjects were deceased at the time this study was conducted. Overall, higher rates of smoking, aerodigestive cancers, and mortality were seen among cases than controls. However, alcohol consumption rates were higher in the control group than in the cases.

HPV DNA was detected in only one of the case samples and none from the control samples tested, a rate of 1010 per 100,000 (95% CI: 30–5500). The HPV genotype detected in the case subject was the oncogenic type HPV16. The patient with the HPV positive OSCC sample was a Caucasian female, aged 62 years at the time of diagnosis of her OSCC. She had an invasive, moderately differentiated SCC of the esophagus and died within six weeks of diagnosis. She had a 45 pack-year history of smoking and was a smoker at the time of her diagnosis. She also had a history of heavy alcohol use.

The pooled analysis with the only other Australian case-control study [22] showed that 9/321 cases and 0/155 controls were positive for HPV. An odds ratio of 9.35 (95% CI: 0.47 to 190.33) was estimated for HPV being a risk factor for OSCC in an Australian population.

## 4. Discussion

Most developed countries are known as low-risk for OSCC due to stable or declining OSCC incidence rates over the last few years. In Australia, the major risk factors for OSCC appear to be smoking and alcohol. Our results suggest that, in this multifactorial cancer, oncogenic HPV may well be a risk factor, but the study was underpowered. The confidence limits around the rate of HPV detection in OSCC in our study ranged from 30 to 5500 per 100,000 cancers, and the rate is consistent with that found in the previous Australian case-control study [22]. The finding of even 1 of 100 cancers positive for HPV may indicate an association, and a larger study would be needed to examine this. The findings are consistent with the only other Australian case-control study, and pooled estimates are suggestive of a risk, but larger studies are needed.

From 1986 to present, there have been only three studies conducted in Australia, to determine whether HPV is a causative factor in OSCC. A variety of methods have been used for HPV detection in OSCC specimens, producing mixed results. The first study carried out in 1986 employed filter in situ hybridization (FISH) methodology and detected HPV in 5/10 (50%) of OSCC specimens and no HPV in macroscopically normal oesophageal tissue biopsied adjacent to the OSCC tumor [24]. Subsequently, Kulski et al. reported 9/39 (23%) of OSCC tissue samples as being positive for HPV, using filter in situ hybridization performed on paraffin-embedded, formalin-fixed tissue (HISTOFISH) [23]. The most recent study by Antonsson et al. was a case-control study using the more sensitive PCR technology, which found 8 out of 222 HPV positive cases, while HPV DNA was not detected in any of the 55 normal oesophageal tissues used as controls [22]. This is an HPV rate of 3603/100,000 cancers, which is in the same range that we found. However, the testing methodology used in this study is different to ours and subject to some limitations which might have resulted in low sensitivity [35]. As such, the HPV detection in that study might have been underestimated.

The pooled analysis of this study with ours and the odds ratio of 9.35 are suggestive of HPV as a risk factor for OSCC but did not reach statistical significance. Clearly, OSCC is a multifactorial cancer, and other risk factors such as alcohol and smoking predominate in countries like Australia. However, the consistency of findings between these two studies and the pooled analysis point, as well as our meta-analysis [21], point to HPV being one of many risk factors for OSCC, even in a low-OSCC incidence country like Australia. In fact, our meta-analysis identified a stronger association of HPV with OSCC in low- and medium-risk countries [21].

Our study adds to the small body of work which currently exists on this topic in the Australian population. In terms of prevalence of HPV in OSCC, there have been some low-risk settings such as USA, Germany, Portuga, and Italy, where high HPV detection rates have been reported in OSCC samples [36–39].

A limitation of this study is the small sample size, and the fact that only one sample was positive for HPV. Studies of OSCC and HPV are difficult and invasive, making large sample size unfeasible. Collection of control specimens entails an oesophageal biopsy, which is an invasive procedure conducted in a healthy subject, and makes it difficult to increase the number of controls to improve statistical power. In fact, our study had almost double the number of controls of the other Australian study [22]. The largest published case-control study globally in our previous meta-analysis had only 265 cases and 357 controls [40]. This makes our study among the largest case-control studies of OSCC and HPV to be conducted. We addressed the small study size by including the pooled analysis with the only other Australian case-control study, which, despite different testing methods (which possibly under-estimated HPV), was done in the same country and was thereby subject to similar environmental and dietary risk factors. The magnitude and direction of the pooled odds ratio, despite not reaching significance, are suggestive, particularly since neither study detected HPV in control tissue, but both studies identified HPV in OSCC tissue. Causality would be supported by finding mRNA in the case samples to show that the virus is transcriptionally active in the tumour tissue.

In summary, our study adds to the very few studies done in Australia, a low-risk country for OSCC. The findings, whilst not conclusive, are consistent with the findings of our meta-analysis and indicate that there may be a role of HPV in OSCC [21]. The recent introduction of universal HPV vaccination for all Australian children, male and female, may have public health benefits beyond the prevention of cervical cancer, and trends in all potentially HPV-related cancers should be monitored over time in the long term. Due to the rarity of OSCC in Australia, long term epidemiologic trends may be the only feasible way of monitoring any role of HPV in this cancer.

## Figures and Tables

**Table 1:** Characteristics of cases and controls.

Variable	Cases (%)*	Controls (%)	*P* value
Mean age	67.5	52.3	N/A
Male	50/99 (51)	52/100 (52)	0.83
Deceased	39/54 (72)	0/100 (0)	<0.001
HPV	1/99 (1)	0/100 (0)	0.31
History of other ADCs	10/58 (17)	0/100 (0)	<0.001
Smoking	55/84 (65)	45/100 (45)	0.005
Alcohol	55/74 (74)	81/100 (81)	0.29

N/A: not applicable; ADCs: Aerodigestive cancers.

*Although a total of 99 case specimens were included in our study, poor quality of obtainable data meant that not all information was recorded for every case. Therefore, variation of denominators in this column indicates the total number of cases for which information on the corresponding variable could be determined.
